# Impact of high-fat diet consumption during prolonged period of pregnancy on placenta structures and umbilical vascular growth in goats

**DOI:** 10.1590/1984-3143-AR2023-0019

**Published:** 2023-05-19

**Authors:** Alessandra Façanha Bezerra, Juliana Paula Martins Alves, César Carneiro Linhares Fernandes, Camila Muniz Cavalcanti, Maria Raquel Lopes Silva, Alfredo José Herrera Conde, Gaby Judith Quispe Palomino, Dárcio Ítalo Alves Teixeira, Aníbal Coutinho do Rego, Ana Paula Ribeiro Rodrigues, Davide Rondina

**Affiliations:** 1 Universidade Estadual do Ceará, Faculdade de Veterinária, Fortaleza, CE, Brasil; 2 Universidade de Fortaleza, Centro de Ciências da Saúde, Fortaleza, CE, Brasil; 3 Universidade Federal do Ceará, Departamento de Zootecnia, Fortaleza, CE, Brasil

**Keywords:** goat, fat diet, pregnancy, fetal growth, oxidative stress

## Abstract

This study aimed to verify the impact of high-fat diet consumption for a prolonged period on oxidative stress, fetal growth, umbilical vascular system, and placental structures in pregnant goats. Twenty-two pregnant goats were grouped into the control diet (n= 11) and fat diet (n = 11). Flaxseed meal was added to the fat diet, replacing the corn grain of concentrate, from gestational day 100 to delivery date. Diets were isonitrogenous and isoenergetic, differing in fat content (2.8% vs. 6.3% dry matter). The fat group showed higher feed intake and total plasma lipid levels than the control group (P < 0.001). No difference was found in placentome, and umbilical vascular development. Fat diet-fed goats exhibited a lower systolic peak in the umbilical artery. At delivery, placental traits were similar with the exception of the cotyledon width (P = 0.0075), which was smaller in the fat group and cotyledon surface (P = 0.0047) for multiple pregnancy of fat diet. Cotyledonary epithelium showed more intense staining of lipid droplets and a greater area for lipofuscin staining in the fat group compared to control group (P < 0.001). The mean live weight of the kids was lower in the fat group in the first week after delivery than in control group. Thus, in goats, the continuous administration of a high-fat diet during pregnancy does not appear to modify the fetal-maternal vascular structures but has an impact on a part of the placental structure; therefore, its use must be carefully evaluated.

## Introduction

During pregnancy, the maternal nutritional profile and blood flow to the fetus are critical for optimal intrauterine development (Lekatz et al., 2018). Physiological changes in umbilical blood flow and thereby, in the transfer of maternal nutrients to the fetus help meet the metabolic needs of the growing fetus. However, presence of excess or restricted maternal nutrition at different times of pregnancy can affect blood flow to the fetus and, consequently, nutrient availability (Vonnahme et al., 2018).

The third trimester of pregnancy is marked by high energy demand associated with lipid mobilization, which increases the transplacental transport of fatty acids (FA) to provide the macronutrients necessary for adequate intrauterine fetal development (Roque-Jiménez et al., 2021). Ewes on feed restriction from gestational days (GDs) 40 to 140 showed high rates of resistance to umbilical blood flow, indicating reduced blood flow to the offspring (Lekatz et al., 2018). High levels of lipids in the maternal diet are associated with abnormal uteroplacental circulation (Frias et al., 2011).

In pregnant women, obesity can affect arterial stiffness and dynamics (Hehir et al., 2009). In addition, resus monkeys fed a high-fat diet demonstrated reduced uteroplacental perfusion associated with a high frequency of late fetal deaths (Frias et al., 2011). Previous studies in mice have shown that the ingestion of high-fat diet during pregnancy is associated with placental remodeling and changes in the maternal intestinal microbiome, promoting changes in lipid metabolism, oxidative stress, and fetal hepatic steatosis (Wang et al., 2021). Thus, the increase in maternal dietary supply of polyunsaturated fatty acids (PUFAs) during late pregnancy may have a direct effect on fetal programming because, during this period, PUFA concentrations increase in fetal circulation (Cetin et al., 2009). Kretschmer et al. (2020) performed a proteomic study in mice fed a high-fat diet and verified the downregulation of cell adhesion genes, basal membrane detachment, and placental lipid accumulation, suggesting alterations in the maternal-fetal transfer function, lipotoxic placental environment, oxidative stress, and fetal metabolic effects.

When it comes to lipid diets in ruminant animals, based on the particularities associated with the type of digestive system and lipid metabolism, dietary formulations normally contain a low concentration of fatty acids (2.5-3.5% of the dry matter [DM] in the diet) (Moallem, 2018). High concentrations of fat (≥ 6% of DM in the diet) can affect the rumen microbiota, efficiency of microbial protein synthesis, and fiber digestion, and may lead to fatal metabolic conditions (Hess et al., 2008). Evidence indicates that high levels of fat in the maternal diet negatively affect the fetal-placental system (Gohir et al., 2019). However, in ruminants, little is known about the effects of including lipids in the diet of pregnant females above the recommended levels, especially for prolonged periods of administration.

Therefore, we hypothesized that the administration of high-fat diet during pregnancy in goats may be an effective feeding protocol for vascular-placental development and maintenance of the doe-kid relationship. Based on this assumption, the present study aimed to verify the impact of diets with high fat content provided for an extended period during late gestation on the metabolic response, oxidative stress, placental structures, and growth of the umbilical vascular system in goats.

## Methods

### Location and animal ethics

This study was carried out at the facilities of laboratory of nutrition and ruminant production (LANUPRUMI) of experimental farm, “Esaú Accioly Vasconcelos,” and School of Veterinary Medicine, Ceará State University, located in Guaiuba, Ceará, in the equatorial zone (4º 2’ 23” S and 38º 38’ 14” W), Brazil. All procedures used in this study were reviewed and approved by the Ethics Committee for Animal Experimentation of Ceará State University (number 05518770/2019).

### Animals and experimental design

In this study, we used 22 pregnant Anglo‐Nubian crossbred adult, pluriparous goats with 41.9 ± 5.1 kg body weight, 2.9 ± 0.1 (score from 1 to 5) body condition score, and age of, 3.4 ± 0.8 years (overall mean ± SD). All animals belonging to the school farm herd had their estruses synchronized, and were naturally mated with Anglo-Nubian bucks. All goats received the same diet composed of a total mixed ration (TMR) based on chopped elephant grass and concentrate. The TMR was prepared in a water solution and furnished to satisfy the nutritional requirements of adult non-dairy goats (NRC, 2007) for each phase of gestation (initial and late gestation) and early lactation.

On GD 100, flaxseed meal was added to the diet, partially replacing corn grain of the TMR concentrate of 11 goats, five with singleton pregnancy, and six with twin pregnancy, together constituting the group fat diet (n = 11). Goats in the control treatment (n = 11) were grouped with the same ratio between pregnancy types (five singleton and six twin) and continued to be fed with a baseline TMR diet, as previously described. The fat diet was offered for 45 days starting on GD 100 till delivery day. The experimental animals were kept in collective stalls grouped according to feeding and pregnancy type, with free access to mineral supplements and water. The TMR was prepared immediately before feeding, and offered twice at 08:00 a.m. and 15:00 p.m. Orts were collected daily and weighed weekly to determine the intake and extent of acceptance of the diet.

### Diets and chemical composition

Food ingredients and the average chemical composition of the diets are presented in Table 1. The diets were isonitrogenous, isoenergetic, and differed in fat content (2.8% vs. 6.3% DM). The proximate composition of the diet samples was determined according to AOAC (1990). Feed, feed orts samples were dried in a forced-air-circulation oven at 55 °C for 72 h and ground in a Wiley mill to pass a 1-mm screen. The samples were analyzed for DM (method 934.01), ash (method 942.05), EE (method 920.39), and crude protein (method 978.04). Neutral detergent fiber was determined using α-amylase without the addition of sodium sulfite (method 973.18), following the recommendation of van Soest et al. (1991). The acid detergent fiber content was determined using the method described by Goering and van Soest (1970). The metabolizable energy (ME) diet content was calculated according to NRC (2007) recommendations: ME (Mcal/kg of DM) = TDN (kg) × 4.4 × 0.82.

**Table 1:** Proportion of ingredients and chemical composition of base diet (Control group) and modified fat diet (g\kg of DM).

	**Diet**	
	**Control**	**Fat**
*Ingredients, g*/*kg of DM*		
Elephant grass	600	600
Ground corn grain	220	120
Soybean meal	140	110
Wheat bran	20	80
Mineral mixturea	20	20
Ground Flaxseed	-	70
*Chemical fraction*		
Dry matter, g/kg as-fed basis	566,7	570.5
Crude protein, g/kg of DM	113.6	116.5
Ether extract, g/kg of DM	28.1	62.6
Ash, g/kg of DM	85.0	86.8
Neutral-detergent fiber, g/kg of DM	507.8	538.2
Acid-detergent fiber, g/kg of DM	298.3	316.9
ME, Mcal/kg of DM	2.32	2.33

^a^Caprine Premix Dourado. Premix containing per kg: Ca 475g, P 35g, K 12.5g, Na 20g, Mg 61.5g, Zn 850mg, Co 15mg, Cu 200mg, S 75mg, F 125mg, I 20mg, Mn 700 mg, Se 7mg, vitamin A 7,500 UI, vitamin D 15,000 UI, and vitamin E 570 UI.

### Fat carcass marker measurement and kid weight

From GDs 100 to 142, weekly abdominal fat depots were estimated by measuring kidney fat thickness behind the thirteenth rib, following the methodology of Härter et al. (2014). Ultrasonography images were taken with a convex transducer at a frequency of 3.5 Mhz (model Z5 Vet; Mindray Bio-Medical Electronics Co., Shenzhen, China), captured in triplicate, and measured using the previously calibrated ImageJ program (Image J, National Institutes of Health, Millersville, MD, USA). During the evaluation, the animals were kept stationary, areas on the right side of the body were shaved, and gel was used as a coupling agent to improve the quality of the images. The offspring were weighed during the first week after delivery.

### Ultrasonography analysis

#### Placentome development and fetal heart rate

**Figure 1:**
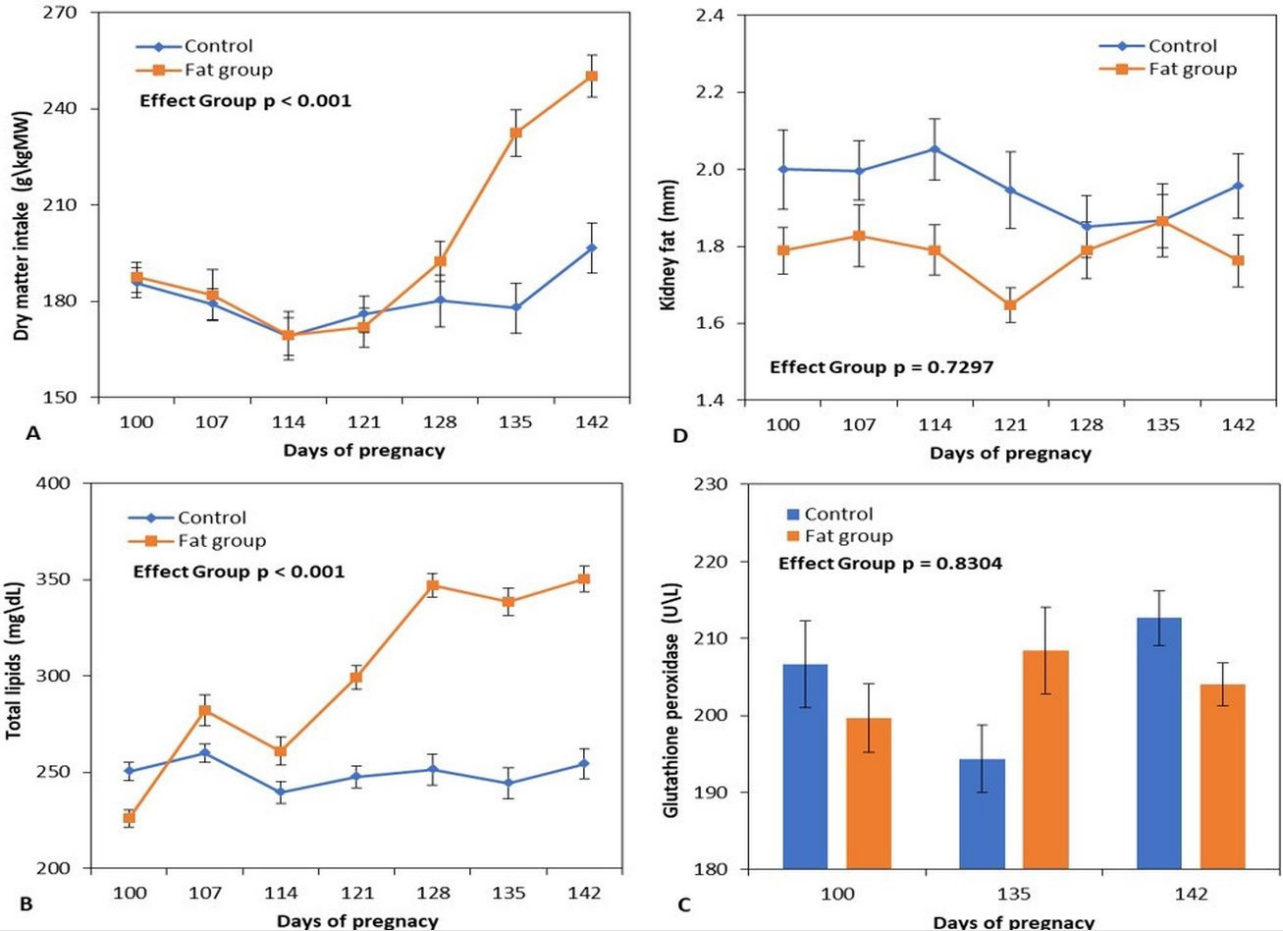
Dry matter intake (A) expressed as g\kg of MW (metabolic weight), plasmatic lipid concentration (B), kidney fat thickness (D) and plasmatic levels of glutathione peroxidase (C), throughout late pregnancy (days 100-145) in goats fed with fat diet (orange mark) or control diet (blue mark). The data was plotted as mean ± SEM. ANOVA *p*-value for diet-group effect was shown in each figure.

Measurements of the placentome through B-mode were performed every 10 days from GDs 100 to 142 using Doppler ultrasonography (Figure 1). The assessments were performed using a transabdominal ultrasound with a 5 MHz convex transducer (Mindray DP 2200 VET, Mindray Biomedical Electronics Co., Shenzhen, China), with goats in a standing position, sweeping the entire abdomen until finding the umbilical cord, and measuring the umbilical cord diameter (Camacho et al., 2018). The average placentome diameter was determined in singleton pregnancies by randomly selecting five placentomes each time umbilical ultrasonography scans were performed. The fetal heart beat was measured using ultrasound equipment previously described in the same assessment interval of the placentome.

### Umbilical vascular development

Every 10 days, from GDs 100 to 142, the diameter of the blood veins and arteries was measured using color Doppler ultrasonography. The assessments were performed using transabdominal ultrasound with a 5 MHz convex transducer (Mindray DP 2200 VET, Mindray Biomedical Electronics Co., Shenzhen, China). In B-mode, the umbilical cord was visualized and the probe was aligned according to the methodology described by Camacho et al. (2018). Using the D-mode, umbilical cord vessel diameters were measured after freezing a cross-sectional image. The vascular structures of the greater caliber were arteries and the smaller structures were veins. For data collection, the average of the two diameters was calculated for each vessel.

### Umbilical artery hemodynamics

Pulsatile waves from the largest artery were recorded in the D-mode in an adequate portion of the umbilical cord, and the hemodynamic parameters determined on GDs 100, 130, and 142 were the peak systolic velocity (PSV), end-diastolic velocity (EDV), resistance index (RI), systolic/diastolic ratio (PSV, EDV), and time-averaged peak velocity (TAP). The scanner settings were calibrated as follows: Doppler sampling frequency = 1.0 kHz, depth = 4.6 cm, and color gain = 60%. During data collection, the average of three separate cardiac cycle waves was calculated from each ultrasound assessment.

### Uterine diameter

In the first and second weeks after delivery, ultrasound images of the uterus were taken to measure the horn diameter. Briefly, the females were kept in a stationary position, and the B-mode ultrasound was assisted by a linear transrectal transducer with a frequency of 5.0 Mhz (DP-2200Vet, Mindray Bio-Medical Electronics Co., Ltd., Shenzhen, China), made rigid by an extension rod, according to the methodology of Degefa et al. (2006). The probe was gently inserted with reference to the cranial edge of the bladder, as a landmark for uterine positioning. Uterine images were captured in triplicate and analyzed using the ImageJ software (ImageJ). The diameter of the uterine horns was measured from the bifurcation, and that of the gravid horn was determined based on the horn with the largest diameter.

### Placenta and cotyledons traits

#### Weight and measurements

At delivery, the placenta was weighed, and the cotyledons were counted. Ten cotyledons for each placenta were sampled to measure their length and width, according to Camacho et al. (2018). Placental efficiency was determined as the ratio between child weight, placental weight, and cotyledonary efficiency as the ratio between child weight and cotyledonary area.

### Cotyledon sampling and staining

At delivery, fragments of cotyledons were immediately collected from the placenta. The lipofuscin staining test was performed with Sudan black dye on a subset of histological slides from the cotyledon sections. Lipofuscin is an intracellular heterogeneous pigment by-product due to failure of cell catabolism, which is conventionally found in the lysosomes or cytosol. The protocol used in this study was modified from that of Ansere et al. (2021). Briefly, the histological slides were dewaxed with xylol, washed in an alcohol gradient until 70% alcohol was reached, and rehydrated with water. After diluting the Sudan Black B (Dinâmica®, SP, Brazil) in 70% alcohol and avoiding its precipitation, a 10 mL syringe with a disc filter was used to drop Sudan black on a clean slide, after which a slide containing the sections was inverted over the Sudan black solution for approximately 2 min. After this procedure, the section slide was separated using tweezers and washed with 50% alcohol and distilled water. The slides were then assembled with glycerol and observed under a light microscope (Eclipse 80i; Nikon, Tokyo, Japan) using 10X and 40X objective lenses. Images obtained were quantified for lipofuscin-positive area using ImageJ Software version 1.54a (NIH, Bethesda, MD, USA) and presented to the total area of the section evaluated.

### Metabolites, β-hydroxybutyrate, and glutathione peroxidase assays

Blood samples were also collected weekly, from GDs 100 to day 142 and in the first week after delivery, using heparinized vacutainer tubes (Labor import, Wei Hai, China) before morning feeding. The samples were centrifuged at 600 × *g* for 15 min, and the plasma obtained was stored at −20 °C for further quantification of metabolites. Plasma concentrations of total protein, glucose, cholesterol, triglycerides, creatinine, urea, glutamic-oxaloacetic acid transaminase (GOT), and glutamic-pyruvic acid transaminase (GPT) were determined using an automated biochemical analyzer (Mindray BS 120, Mindray®) and commercial kits (Bioclin, Quibasa, Minas Gerais, Brazil). The sensitivity of the assay kit was 0.043 g/dL for total protein, 1.5088 mg/dL for glucose, 1.472 mg/dL for cholesterol (CHL), 2.58 mg/dL for triglycerides (TRY), 1.51 mg∕dL for urea, 0.135 mg/dL for creatinine, 2.874 U/L for GOT, and 0.998 U/L for GPT. Total lipids (TL) were calculated using the equation: TL = 2 × (CHL + TRY) × 1.1. Glutathione peroxidase and β-hydroxybutyrate (BHB) were analyzed using a semi-automatic biochemical analyzer (Randox RX Monza TM, Randox Laboratories, Crumlin, UK) and commercial kits (Randox Laboratories) with a sensitivity of 75 U/L for GPx and 0.100 mmol/L for BHB.

### Statistical analysis

Statistical analyses were performed using Statistica Software, version v. 13.4.0.14 (2018; TIBCO Software, Inc., Palo Alto, CA, USA). Data were initially verified for mathematical assumptions by Kolmogorov-Smirnov and Bartlett tests, and when these conditions were not respected, the transformation in log10x was applied.

Data were subjected to an analysis of variance (ANOVA) of the GLM procedures in a factorial arrangement. For feeding intake, metabolite levels, hemodynamic parameters of the umbilical artery, and fetal heart rate, the main effects tested were the diet group (control, fat diet), the effect of interval of assessment used (time), type of pregnancy (singleton or twin), and interaction diet vs. time and diet vs. type of pregnancy. For placental traits and parameters measured after delivery, the fixed effects tested were diet group and type of pregnancy (singleton or twin) and the interaction between diet and type of pregnancy. Lipofuscin staining of the cotyledon area was performed to determine the diet group effect. Descriptive ultrasonography data (kidney fat thickness, placentome diameter, and umbilical vein and artery diameters) were analyzed using the GLM procedures for repeated measures of ANOVA. The factors used in the model included diet, time, type of pregnancy, and the interaction of diet vs. time and diet vs. type of pregnancy. The recorded anatomic images (1, 2, and 3) were repeated measures. All pairwise comparisons of the means were performed using the Newman-Keuls test.

## Results

### Feeding intake and fat mass

A significant effect of diet (P < 0.001) was observed on DM intake (Figure 1A), expressed per kg of metabolic weight (MW) (178.5 ± 2.6 g\kg MW, control group vs. 193.4 ± 3.1 g\kg MW, fat group). The fat group showed an increase in food consumption from GD 128 (Figure 1A), indicating a significant interaction between diet and diet administration interval (P < 0.001).

During the experimental period (GDs 100-142), no significant differences (P = 0.7297) were observed between the food groups in terms of kidney fat thickness (Figure 1D) and no significant interactions.

### Metabolites, glutathione peroxidase and BHBA

A significant effect (P < 0.001) of diet in relation to total plasma lipids (240.7 ± 5.6 mg\dl, control group vs. 280.2 ± 7.5 mg\dl, fat group) was observed, recording an increase in the fat group from GD 114 (Figure 1B) and a significant interaction between the measurement range and food group (P = 0.0098).

No differences between the groups in the levels of glutathione peroxidase (P = 0.8304; Figure 1C), glucose (P = 0.5933; Table 2), total proteins (P = 0.2664; Table 2), urea (P = 0.2467; Table 2), or glutamic-pyruvic acid transaminase (P = 0.7320; Table 2) were found. The fat diet led to an increase in glutamic-oxaloacetic acid transaminase (P = 0.0059) and reduction in creatinine (P = 0.0010) and BHBA (P = 0.0037) levels.

**Table 2:** Metabolic effort, and kidney and liver injury throughout the late pregnancy (gestational days 100-145) in goats fed with a fat diet or control diet. Values are shown as means ± SEM.

**Parameters**	**Day** *	**Diet**			**p Value**				
		**Control**	**Fat**	**SEM**	**Diet**	**Time**	**TP**	**D vs T**	**D vs TP**
*Does exposed, n*		11	11						
*Metabolic effort*									
Glycose, mg\dl S	100-142	51.7	52.2	0.98	0.5933	0.3584	0.0050	0.6591	0.1713
Total protein, mg\dl	100-142	5.9	5.7	0.08	0.2664	0.2995	0.0283	0.0694	0.9570
BHB, mmol\L	100-142	0.39	0.30	0.02	0.0037	< 0.001	0.0205	0.0821	0.4896
*Kidney injury*									
Creatinine, mg\dl	100-142	0.7	0.6	0.02	0.0010	0.0087	0.9786	0.1298	0.1028
Urea, mg\dl	100-142	26.9	30.3	1.38	0.2467	< 0.001	0.0203	0.0067	0.5899
*Liver Injury*									
GOT, U.L	100-142	93.0	113.5	4.10	0.0059	< 0.001	0.3711	0.0896	0.2474 474
GPT, U.L	100-142	24.2	25.1	1.11	0.7320	0.0404	0.7004	0.1969	0.7087

* Gestational day when measurement was performed. GOT: glutamic-oxaloacetic acid transaminase; GPT: glutamic-pyruvic acid transaminase; Time, ANOVA effect for interval of assessment used; TP, ANOVA effect for type of pregnancy (singleton or twin pregnancies).

Additionally, a significant interaction was found for the concentrations of urea in the fat diet between group and interval (P = 0.0067; Table 2), as results of the greater increase of plasmatic urea occurred during the gestational interval (22.6 ± 2.3 mg\dl, GD 100 vs. 38.1 ± 2.2 mg\dl, GD 142).

### Fetal and vascular development and hemodynamic parameters

All groups showed a significant increase (effect of time, P < 0.01) during the gestational interval analyzed in relation to the diameter of the umbilical arteries and their hemodynamic parameters, with the exception of the resistance index and PSV\EDV rate, which reduced their values (Table 3). In addition, the fetal heartbeat decreased with the measurement range (P = 0.0038). The animals in the fat group had a lower systolic peak velocity and time-averaged peak velocity (P < 0.05) than those in the control group (Table 3). No significant interactions were observed.

**Table 3:** Fetal heart rate, umbilical blood vessels system and hemodynamic Doppler parameters of umbilical artery throughout late pregnancy (gestational days 100-145) in goats fed with a fat diet or control diet. Values are shown as means ± SEM.

**Parameters**	**Day** *	**Diet**			**p Value**				
		**Control**	**Fat**	**SEM**	**Diet**	**Time**	**TP**	**D vs T**	**D vs TP**
*Does exposed, n*		11	11						
Fetal heart rate, bpm	100-142	138.4	146.1	4.11	0.7895	0.0038	0.1790	0.2340	0.9592
*Umbilical vascular development*									
Vein diameter**, cm	100-142	11.5	11.1	0.27	0.8442	0.4225	0.4204	0.5153	0.2146
Artery diameter**, cm	100-142	11.1	10.7	0.25	0.5423	0.0074	0.4991	0.7756	0.7625
Total vascular area, cm^2^	100-142	315.3	364.1	24.5	0.1727	0.1709	0.8806	0.5723	0.0652
*Umbilical artery hemodynamic*									
Peak systolic velocity (PSV), cm\s	100-142	58.7	45.4	2.32	0.0153	< 0.001	0.0221	0.7522	0.5129
End-diastolic velocity (EDV), cm\s	100-142	25.0	19.1	1.31	0.2251	< 0.001	0.0439	0.6263	0.6463
PSV\EDV	100-142	2.5	2.5	0.08	0.6568	0.0112	0.7288	0.7036	0.8579
TAP***, cm\s	100-142	37.2	28.1	1.72	0.0144	< 0.001	0.0073	0.7258	0.3694
Resistance index	100-142	0.6	0.6	0.01	0.6330	0.0150	0.1982	0.8909	0.7937

* Gestational day when measurement was performed; **Sum of vein or artery diameters; ***Time-averaged peak velocity. Time, ANOVA effect for interval of assessment used; TP, ANOVA effect for type of pregnancy (singleton or twin pregnancies).

### Placental traits

**Figure 2:**
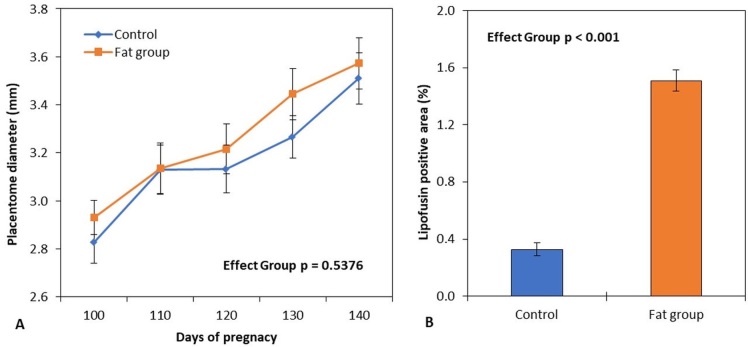
Placentome diameter throughout late pregnancy (days 100-145) and lipofuscin area of placental cotyledons at delivery in goats fed with fat diet (orange mark) or control diet (blue mark). The data were plotted as mean ± SEM. ANOVA *p*-value for diet-group effect was shown in each figure.

We observed no significant effect of food group (P = 0.5376), and no significant interaction in relation to the diameter of the placentome (Figure 2A), which showed significant growth in both groups during the experimental period (P < 0.001).

**Figure 3:**
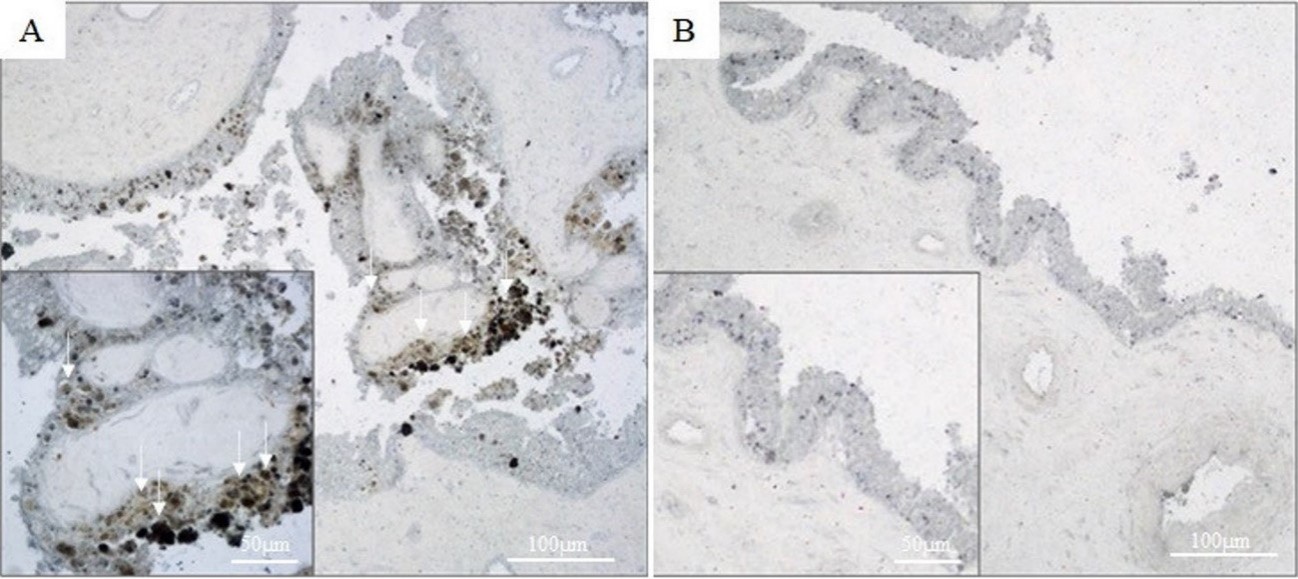
Lipid droplets (white arrows) colored by Sudan Black staining in placental cotyledons collected at delivery in goats fed with a fat diet (Left **figure A)** or control diet (Right **figure B).** Representative region of cotyledonary epithelium stained with Sudan Black was shown.

At parturition, the main placental parameters did not differ (P > 0.05) between the diets (Table 4), except for the cotyledon width (P = 0.0075), which was lower in the fat group. Interactions (P < 0.01) were also observed between the type of pregnancy and diet group where multiple parturition of the fat diet showed a lower cotyledon width (4.3 ± 0.1 cm vs. 3.2 ± 0.1 cm; P < 0.001) and cotyledon surface (9.8 ± 0.6 cm^2^ vs. 7.6 ± 0.4 cm^2^; P = 0.0047) than those of the control group. Histological analysis of the cotyledonary epithelium showed more intense staining of lipid droplets in the fat group (Figure 3) and a greater (P < 0.001) area for lipopigment lipofuscin staining (Figure 2B) than those of the control group.

**Table 4:** Placental traits at delivery and metabolic, uterine diameter and kid’s weight measured after delivery in goats fed with a fat diet or control diet. Values are shown as means ± SEM.

**Parameters**	**Diet**			**p Value**		
	**Control**	**Fat**	**SEM**	**Diet**	**TP**	**D vs TP**
Does exposed, n	11	11				
*Reproductive traits at delivery*						
Days of pregnancy, day	145.4	145.6	0.13	0.4954	0.4954	0.7323
Placental weight, g	0.5	0.5	0.03	0.5942	< 0.001	0.2531
Placental efficiency	6.9	5.9	0.53	0.2777	< 0.001	0.5327
No. of cotyledons, n	90.8	103.2	5.40	0.4296	0.1498	0.1115
Cotyledon length, cm	2.8	2.8	0.13	0.9308	0.1896	0.2768
Cotyledon width, cm	3.4	3.1	0.08	0.0075	< 0.001	< 0.001
Average cotyledon surface area, cm^2^	7.3	6.7	0.29	0.4210	< 0.001	0.0010
Cotyledon efficiency	108.8	92.4	8.32	0.5171	< 0.001	0.5878
Total cotyledon surface area, cm^2^	305.2	307.3	10.81	0.8851	< 0.001	0.7786
*Response after delivery* *						
Kids body weight, kg	3.0	2.6	0.10	0.0401	< 0.001	0.2558
Shrinkage rate of uterine diameter**, %	21.4	26.0	3.89	0.2383	0.2710	0.7577
BHB, mmol\L	0.34	0.30	0.03	0.3620	0.2216	0.8477
Total lipids, mg\dL	202.4	223.6	14.5	0.3010	0.5224	0.8546
Glutathione peroxidase, U\L	221.8	220	5.61	0.8421	0.6619	0.5747

* Measurements performed one week after delivery; **Difference between uterine diameters in 2nd and 1st weeks after delivery. TP: ANOVA effect for type of pregnancy (singleton or twin pregnancies).

### Response after delivery

Table 4 describes the main results of the responses of the diet groups recorded in the first week after delivery. The fat group showed lower mean live weight of the children than the control group (P < 0.05). Plasma concentrations of BHB and total lipids were similar between the groups, with no interaction between the type of pregnancy and diet. The rate of uterine diameter reduction measured between the 1st and 2nd postpartum weeks was similar between the groups.

## Discussion

Maternal nutrition is the main source of FA in the developing fetus during pregnancy. In the third trimester, as there is an increase in LC-PUFA in the fetal circulation, the availability of nutrients via maternal nutrition can impact the development of the offspring (Cetin et al., 2009). Despite the importance of PUFAs for fetal development in ruminants, the transport of FA via the cotyledonary placenta is limited, which raises an important question regarding the effect of lipid inclusion in the diet of the mother during pregnancy (Roque-Jiménez et al., 2021). Furthermore, fetal-placental vascularization may be affected by nutritional restriction as a compensatory mechanism in an attempt to meet fetal metabolic demands (Lemley et al., 2018).

The main findings of the present study showed that diets with similar energy and high fat contents can be used during pregnancy for prolonged periods. Although the addition of fat induced dyslipidemia during the experimental period, metabolic and glutathione peroxidase indicators did not show specific stress states on the part of the animals, except for GOT levels. In contrast, the fat group showed an evident increase in food consumption in the last two weeks before kidding. In addition to metabolic changes, lipid diet generally has negative effects on dry matter intake (Oliveira et al., 2018). Decreased DM intake can be expected when dietary fat concentration exceeds 6% (Martin et al., 2010). However, Nogueira et al. (2019) added 8% fat concentration to cow feed and did not observe any difference in DMI between treatments. According to Joy et al. (2021), increasing the lipid content of the diet of beef heifers improves palatability and feed efficiency and increases the energy density of the diet. In the present study, the microbial capacity to saturate unsaturated fatty acids did not exceed, resulting in regular microbial digestion and intake. The mechanisms of intake reduction caused by lipid supplementation are related to the biohydrogenation of unsaturated fatty acids in the rumen.

Lipid levels and the type of dietary fatty acids influence blood biochemical parameters. Therefore, they are sensitive indicators of health status and reflect the intensity of metabolic processes in animals (Adeyemi et al., 2016). According to Tosto et al. (2021), high concentrations of GOT in dairy goats are involved in liver injury, as these enzymes do not occur naturally in the plasma circulation. However, during the transition period in ruminants, an increase in GOT occurs naturally, which can be intensified by a lipid diet, due to the greater accumulation of hepatic triglycerides, to meet the energy demands of the period (Thrall et al., 2012; Tharwat et al., 2015). Furthermore, previous studies have reported that goats fed flaxseed tend to have higher levels of GOT, which may be related to the higher liver turnover rate in these animals (Nudda et al., 2013).

Excessive use of dietary fat during pregnancy can be harmful to the maternal placental system and fetal development. However, little is known about the limits of inclusion in ruminants, especially due to the inhibitory modulation of the rumen environment and its functionality, as well as the difficulty in interpreting lipid metabolism due to the remodeling of dietary lipids at the rumen level (Roque-Jiménez et al., 2021). Our results showed that the presence of fat in the diet did not induce changes in the development of the umbilical vascular system or placental structure. However, the lower expression of some of the hemodynamic parameters of the umbilical artery (PSV and TAP), together with the reduction in the cotyledonary surface in multiple pregnancies and in the birth weight of the offspring, leads to a cautious consideration of the real impact of this type of diet during pregnancy.

The PSV and TAP are the preferred parameters for assessing placental blood flow volume in humans (Liu et al., 2021). The negative impact on this parameter may be associated with higher vascular impedance, decreased blood flow to the fetus, and lower fetal viability (Elmetwally and Meinecke-Tillmann, 2018), which in turn may explain the birth weight of fetuses of mothers fed lipids. According to (Lin et al., 2019), rats fed lipid diets have reduced placental weight and fetal growth restriction due to alterations in the placental renin-angiotensin system, which regulates uteroplacental blood circulation and has been associated with maternal hypoxic stress. Changes in the intake and metabolism of nutrients promote changes in fetal-placental circulation, making this a key issue associated with optimal fetal development because they are related to the supply of nutrients to the fetus (Beltrame et al., 2017). Previous studies in ruminants have shown that the maternal nutritional profile can affect uteroplacental blood flow (Camacho et al., 2018).

The ability of the placenta to provide nutrients to the fetus is determined by many factors, including the mother's nutritional status, uteroplacental blood flow, and expression and function of trophoblast nutrient transporters (Cleal et al., 2022). However, the results regarding the effect of lipids on the placenta and fetus are still controversial, and this nutrient may reduce (Lin et al., 2019), increase (Jones et al., 2013) or not change (Rosa Velazquez et al., 2020) the size of the fetus and placenta. In goats, the maternal nutritional profile can affect placental characteristics and proteomes at different times of pregnancy (Yan et al., 2018). The size and surface area of cotyledonary structures are the most influential placental characteristics on maternal-fetal exchange and placental efficiency and are related to the weight and health of offspring (Ocak et al., 2018).

In ruminants, the transplacental transport of free fatty acids from the mother to the fetus can occur either by simple diffusion or by specific transporters (Roque-Jiménez et al., 2021). In our study, we identified a greater accumulation of lipid droplets in the cotyledon tissue of the DLD group, which can be explained by the increased transport of AG to the cotyledons that activate PPARg, a lipid-activated transcription factor (Calabuig-Navarro et al., 2017). In turn, PPARg stimulates the transcription of key genes involved in lipid esterification and storage, such as DGAT1, SCD1, and ACC (Calabuig-Navarro et al., 2017). This storage is thought to help protect the fetus from excess maternal lipids by diverting them to esterification and storage, rather than transport to the fetus and oxidation (Lewis and Desoye, 2017). (Zhu et al., 2010) demonstrated that cotyledons from placentas of obese ewes during late pregnancy had lower expression of AG 4 transporter protein (FATP) than those of ideal weight. FATP4 is involved in the transport of AG into fetal circulation, indicating that there is less transport of AG to the fetus. This mechanism is believed to protect the fetus from lipotoxicity (Lewis and Desoye, 2017).

Adverse intrauterine nutritional exposure as a consequence of maternal nutrition at different times of pregnancy in ruminants can have both short- and long-term consequences for offspring, a phenomenon known as fetal programming (Roque-Jiménez et al., 2021). The placenta is intimately involved in this phenomenon, as it is able to adapt to help the fetus grow, develop, and deliver the nutrients necessary for this to happen optimally (MacCannell et al., 2015). Thus, restriction or excess maternal nutrients can lead to physiological, metabolic, and epigenetic changes in the placenta, which can affect offspring development (Lesseur and Chen, 2018).

## Conclusion

In conclusion, the prolonged use of a high-lipid diet during pregnancy in goats does not induce modulation of the umbilical vascular system, but partially alters the placental cotyledonary structure. Therefore, the authors suggest a careful evaluation of the use of this nutritional protocol and encourage further studies with the aim of providing more information to support the effectiveness of the use of fat for longer periods during pregnancy in goats.
